# Tailoring Evaluations of Chronic Rhinosinusitis: Understanding Sleep and Its Effect on Memory Through Actigraphy

**DOI:** 10.3390/jpm15060249

**Published:** 2025-06-14

**Authors:** Donyea Moore, Rachel Nolte, Yitong Huang, Shreya Maharana, Pavan Nataraj, Bichun Ouyang, Mahboobeh Mahdavinia

**Affiliations:** 1 Division of Allergy and Immunology, Department of Internal Medicine, UT Health Houston, 6410 Fannin St, Houston, TX 77030, USA; donyea.moore@uth.tmc.edu (D.M.);; 2 Division of Allergy and Immunology, Department of Internal Medicine, Rush University Medical Center, 1725 W. Harrison St, Chicago, IL 60612, USA; shreyamaharana8@gmail.com (S.M.);; 3 Department of Mathematical Sciences, Smith College, 10 Elm St, Northampton, MA 01063, USA; yhuang86@smith.edu; 4 Department of Neurological Sciences, Rush University Medical Center, 600 S. Paulina St, Chicago, IL 60612, USA; bichun_ouyang@rush.edu

**Keywords:** actigraphy, chronic rhinosinusitis, cognition, inflammation, sleep, personalized medicine

## Abstract

**Background/Objectives:** Chronic rhinosinusitis (CRS) is a persistent inflammatory condition of the sinonasal mucosa lasting for at least three months. For patients, CRS-related sleep disturbances can significantly disrupt circadian rhythms, leading to further health complications such as cognitive impairment. Despite the well-documented sleep disturbances associated with CRS, there is limited research on objective assessment methods. Additionally, the severity of these issues can vary among patients. This study aims to assess sleep quality and timing in CRS patients and investigate their impact on cognition, providing guidance for personalized and tailored assessment and management of CRS. **Methods:** Our case–control study compares sleep patterns and cognitive function between CRS patients and healthy controls utilizing actigraphy, a non-invasive device for measuring sleep–wake cycles and circadian rhythms. The actigraphy-derived sleep variables include inter-daily variability, intra-daily variability, highest 10 h activity (M10), lowest 5 h activity (L5), relative amplitude (RA), sleep onset latency, sleep efficiency, sleep and wake time, time spent in bed, total sleep time, and wakefulness after sleep onset. We also used a standard questionnaire assessing sleep quality, the Pittsburgh Sleep Quality Index (PSQI). **Results:** Our study enrolled 44 CRS and 43 control participants. Our findings indicate that the actigraphy-derived sleep variables were comparable between groups, all with a *p*-value > 0.05. However, CRS patients exhibited greater early morning activity and significantly lower PSQI-reported sleep quality compared to controls (8.78 ± 3.45, 4.71 ± 2.96, respectively; adjusted *p* < 0.001). Actigraphy-derived sleep variables showed trends towards significance in association with episodic memory (*p* = 0.051) and executive function (*p* = 0.15). **Conclusions:** Actigraphy-derived sleep outcomes revealed associations with episodic and executive function, underscoring the potential of actigraphy in understanding the individualized sleep-related cognitive impacts in CRS patients. This highlights the importance of personalized assessment and management strategies to address the unique sleep and cognitive challenges faced by each patient.

## 1. Introduction

Chronic rhinosinusitis (CRS) is a persistent inflammatory disorder that predominantly affects the sinonasal mucosa, leading to prolonged inflammation lasting for a minimum of three months [1]. CRS is categorized into two subtypes, CRS with nasal polyps (CRSwNP) and CRS without nasal polyps (CRSsNP). This condition affects approximately 4.5 to 12% of the global population [1,2]. CRS represents a significant public health concern, as it has a negative impact economically and is associated with considerable impairment in patients’ productivity and quality of life [3]. One of the most consequential aspects of CRS is the associated sleep disturbance [4]. Sleep disturbance can result in disruption of the circadian rhythm, which can further exacerbate a range of health complications, including cognitive dysfunction, metabolic abnormalities, and cardiovascular diseases [5]. In addition to sleep disturbances, cognitive impairments, particularly in memory, attention, and executive function, are increasingly reported among individuals with CRS [6,7]. Studies have shown that lower Wake After Sleep Onset (WASO) scores are associated with better memory performance [8]. These deficits can have profound effects on daily functioning, work performance, social engagement, and overall independence, highlighting the importance of sleep continuity for cognitive health [3,4]. Factors such as disruptions of sleep and circadian rhythms, which are frequently observed in CRS patients may aggravate cognitive dysfunction [9]. This relationship between sleep and cognition indicates that interventions targeting sleep quality may have promising benefits for cognitive function. While reported sleep disturbances in CRS patients are well-documented, research on the use of objective methods to assess sleep quality, efficiency, and timing is limited. Actigraphy is a non-invasive, wrist-worn monitor that collects data on sleep–wake cycles and has shown to be a promising tool in assessing sleep in individuals with chronic inflammatory conditions, highlighting its potential for personalized medicine [10,11].

This study seeks to test the utility of actigraphy, a novel and non-invasive tool, in assessing the sleep/wake cycle and circadian rhythm in CRS patients. By investigating the role of sleep disturbances and timing shifts measured by actigraphy on cognition, this study aims to provide personalized insights and tailored management strategies for CRS patients. Importantly, not all CRS patients experience these problems to the same degree; some have more severe forms of the condition. Therefore, it is crucial to assess and treat each patient individually based on their specific symptoms.

## 2. Materials and Methods

Our case–control study aimed to compare the sleep quality and circadian rhythms of patients with chronic rhinosinusitis (CRS) to those of healthy controls. We assessed sleep patterns using both objective and subjective measures and tested whether these changes impacted the individual’s cognitive function. The study was approved by the Institutional Review Board (IRB) and conducted at a tertiary care academic center.

### 2.1. Inclusion and Exclusion Criteria

CRS subjects and healthy controls were recruited and consented if they were between the ages of 18 and 75 years, had a BMI ≤ 35, did not have a medical history of cancer diagnosis within 3 years prior to enrollment, and did not have sleep or cognitive conditions such as sleep apnea or other neurologic diseases impacting sleep. Healthy individuals with no allergy-related health issues and similar demographics were included as controls.

Participant data was collected through surveys completed by patients, thorough review of their electronic medical records, and 1-week actigraphy assessments as detailed below.

Actigraphy: To measure circadian rhythms, we utilized the Actiwatch 2; Philips^®^ [12]. This wrist-worn activity monitor is designed to record data relevant to circadian rhythms and sleep timing parameters. The Actiwatch 2^®^ features a solid-state “Piezo-electric” accelerometer with a bandwidth of 0.35–7.5 Hz and a sensitivity of 0.025 G. It also includes a light sensor with an accuracy of 10% at 3000 Lux and a measurement range of 5–100,000 Lux. Participants wore the Actiwatch 2^®^ snugly on their non-dominant wrist for 7 days, allowing us to collect comprehensive data on their physical motion and light exposure during the week on both workdays and free days, which are critical for analyzing circadian rhythms. Participants were given a brief training session to explain how to wear the Actiwatch, complete their sleep diaries accurately, and troubleshoot common device issues. In addition to wearing the Actiwatch 2^®^, participants were asked to keep a sleep diary where they recorded the time they went to bed, woke up, took naps, and when they removed and replaced the device for any reason.

Sleep quality: We used a standard questionnaire assessing sleep quality, the Pittsburgh Sleep Quality Index (PSQI). The PSQI questionnaire consists of seven component scores, each ranging from 0 to 3, with higher scores indicating greater disturbance. The seven components are sleep quality, sleep latency, duration, sleep efficiency, disturbances, use of medication, and daytime dysfunction. These component scores are summed up to obtain a global PSQI score, where higher scores represent poorer overall sleep quality. PSQI is a commonly utilized assessment to assess sleep quality. PSQI was administered at baseline during the in-person visit. Sleep diaries were used to supplement both subject and objective data. Diary data provided insight into subjective perception of sleep onset. All data was linked to individual records in a secure REDCap database.

Rest–activity data were analyzed using standard nonparametric method to obtain inter-daily stability, intra-daily variability, relative amplitude, and midpoint of the most active 10 h period, and the least active 5 hr period in the average 24 h pattern.

Cognitive function of all participants was assessed by conducting cognition batteries across six cognitive domains, which included Episodic Memory, Attention/Processing Speed, Visuospatial Abilities, Semantic Memory, Working Memory, and Executive Function (10). Cognitive tests were administered in a quiet, distraction-free setting by trained research staff. Tests were chosen based on standardized batteries with known reliability. Tests included: Episodic Memory: (1) Word-list recall tasks (immediate and delayed), (2) Attention/Processing Speed: Digit Symbol Substitution and Number Comparison tests, (3) Visuospatial Abilities: Progressive Matrices and Line Orientation tasks, (4) Semantic Memory: Boston Naming Test, Verbal Fluency, and Category Fluency, (5) Working Memory: WAIS Digit Span Forward/Backward/Ordering, (6) Executive Function: Stroop Color and Word test, the National Adult Reading Test (NART), and Wechsler Adult Reading Test (WART).

### 2.2. Statistical Analysis

Continuous variables were presented as the mean with a standard deviation, and categorical variables were presented as frequencies with percentages. To compare variables between two groups, we used two-tailed t-tests for normally distributed continuous variables, non-parametric Wilcoxon rank-sum tests for nonnormally distributed continuous variables, and Chi-squared tests for categorical variables. For all descriptive groupwise comparisons, raw (unadjusted) *p*-values are provided, and we report both significant and nonsignificant *p*-values. For time-varying activity variables, one of our main interests was the pattern of difference over time. Therefore, we tested groupwise differences using a generalized estimating equations (GEE) model, which can account for both within-subject and between-subject variance using an interaction term between group (i.e., CRS vs. Control) and time. Statistical significance was set as a two-sided *p*-value < 0.05.

Bivariate associations between the cognition variables and sleep measures were tested. For all bivariate associations, unadjusted *p*-values and Spearman correlation coefficients are reported. Sleep variables that met the significance threshold (unadjusted *p* <0.05) in bivariate association testing were sequentially entered into multivariable models including a priori covariates of age, body mass index and years of education to test independent associations with corresponding cognition variables. Statistical analyses were conducted using R version 4.4.0.

## 3. Results

**Figure 1:**
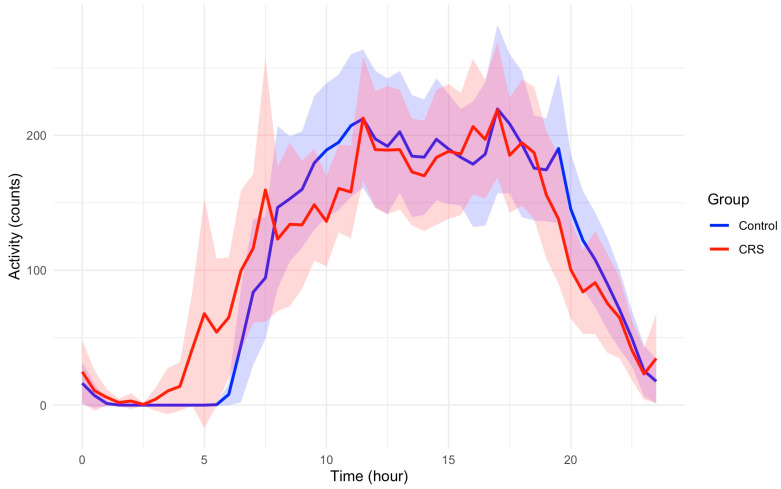
The 24 h activity profiles of participants in control and CRS. Average hourly activity counts are plotted across a 24 h period relative to clock time. Shaded areas represent 95% confidence intervals. Participants with CRS have greater early morning activity compared to those in controls. Peak activity counts are not different between two groups.

The study included 87 participants who had multiple days of actigraphy recording and completed cognitive function assessments, comprising 44 participants with CRS and 43 control participants. Table 1 reports the demographic characteristics by study group. While the actigraphy-derived sleep variables such as Inter-daily Stability, Intra-daily Variability, and Relative Amplitude are comparable between the two groups (Table 2), participants with CRS have greater early morning activity compared to those in controls. Figure 1 demonstrates activity profiles across a 24 h period between control and CRS groups.

It is noteworthy that the overall activity patterns in CRS were not significantly different from controls in GEE models. However, the sleep quality measured by PSQI was significantly lower among CRS patients compared to controls with a mean (SD) of 8.78 (3.45) vs. 4.71 (2.96) in CRS and controls, respectively; adjusted *p* < 0.001.

CRS subjects had lower raw scores in processing speed, executive function, and semantic memory in crude analyses. However, these differences were not statistically significant after adjusting for age, sex, education, race, and BMI (Table 3). The results of the bivariate association tests between the six cognition measures and actigraphy-derived sleep variables are provided in Table 4. The following associations met an unadjusted significance threshold in bivariate analyses: (1) worse episodic memory was associated with longer wake after sleep onset (WASO); and (2) better executive function was associated with higher daytime activity (M10). To further test whether actigraphy-derived sleep variables are associated with different domains of cognition, we used multivariable models adjusted a priori for age, BMI, education, sex, and race. We then sequentially introduced the significant bivariate predictor variables, WASO and M10 into these models. In multivariable models, WASO had a trend towards significance in association with episodic memory (*p* = 0.051), while M10 was no longer significantly associated with executive function (*p* = 0.15).

## 4. Discussion

Previous studies, including ours, have shown an undeniable disruption of sleep quality in CRS compared to healthy controls, which is also confirmed by the current finding of significantly decreased PSQI [4]. However, as it appears in the current analyses, these disruptions are not impacting all aspects of sleep and its circadian associations. One positive finding is the relatively greater early morning activity between 3 and 7am in CRS patients compared to healthy controls, as depicted in Figure 1. This might be explained by the circadian rhythm of inflammatory pathways [13]. The higher amplitude of systemic inflammatory effect in respiratory tissue controlled by the circadian rhythm results in early morning symptoms with a peak of activity around 4am [14]. This is observed in other T-helper 2-driven diseases like asthma which tend to have increased symptoms during the early morning hours [14,15]. It is noteworthy that this effect is not explained by the use of corticosteroids and is a function of the circadian rhythm control in these patients [16]. A similar phenomenon might be the underlying cause of observed increased early morning awakening and movements (before the patient is ready to wake up) in CRS patients.

Sleep disturbances are associated with poorer cognitive performance in healthy individuals [9]. In our current study, after adjusting for multiple potential confounding factors, poor episodic memory had a trend toward association with less continuous sleep, measured by wake-after-sleep onset (WASO). The findings from a recent meta-analysis indicate that longer WASO is positively correlated with poorer episodic memory performance in older adults (those above the age of 60) [17]. Specifically, WASO showed a stronger correlation with memory performance in older adults compared to other sleep parameters such as total sleep time, time spent in slow-wave sleep, and time spent in REM sleep [17]. This data along with previous findings emphasizes the role of sleep efficiency on memory in older adults, including those with CRS [18]. Sleep plays a crucial role in replenishing the ability to learn and recall information [19,20]. Lack of efficient sleep can significantly impair memory retention and recall [19]. Another important point is that sleep and circadian parameters change in an individual during the aging process [21]. Furthermore, chronic inflammation, which is the hallmark of disease in CRS [22], goes hand in hand with sleep and circadian abnormalities through a bidirectional path [13]. As mentioned above, the association of WASO and memory is age-dependent and more prominent in adults over 60, which is of particular importance in a condition like CRS which is mostly adult onset with a higher incidence in older adults [23]. These findings suggest a potential difference in how sleep disturbances affect cognition in CRS patients as they age, raising important questions about accelerated sleep and circadian disruption and its potential link to memory in the context of chronic inflammation. As CRS patients age, the severity of these disruptions may vary, highlighting the need for individualized assessment and treatment. Longitudinal studies examining the cumulative impact of circadian rhythm disturbances on cognitive function in CRS patients may provide a clearer understanding of how long-term disruptions might contribute to cognitive decline.

Our study has several limitations. One key limitation is the small number of this pilot study. As mentioned, the observed trend, though close to significance (*p* = 0.051), could be due to the low power of the study and needs confirmation. Additionally, there were significant differences in age and sex between the two groups, which may have introduced confounding effects. However, these demographic disparities were considered when interpreting the results. Furthermore, the CRS patients were not stratified based on their previous medication regimen, which could affect our results. The medications used for treating CRS may improve symptoms, thereby enhancing sleep quality and potentially lowering WASO scores. This therapeutic effect could influence the observed associations between sleep and cognitive outcomes. Larger prospective and long-term studies, including a broader range of comorbidities, are needed to validate our findings and explore the impact of comorbid conditions on the relationship between sleep, cognition, and CRS. Studies, including a broader range of comorbidities, are needed to validate our findings and explore the impact of comorbid conditions on the relationship between sleep, cognition, and CRS.

In conclusion, this study provides important insights into the complex relationship between CRS, sleep disturbances, and cognitive function. The observed trends in our study highlight the potential for compensatory mechanisms and treatment effects to influence cognitive outcomes. Future research should explore these mechanisms further, particularly the role of inflammation, medication, and compensatory strategies in modulating cognitive function in CRS patients. Understanding the interplay between these factors is crucial for developing targeted interventions to mitigate cognitive decline and improve the quality of life for individuals living with CRS. Personalized approaches to monitoring and managing these disturbances are essential to address the unique needs of each patient.

## Figures and Tables

**Table 1:** Demographics and clinical characteristics of 44 CRS and 43 control participants.

Demographic and Clinical Characteristic Variables	Control	CRS	*p*-Value ^a^
Age (Mean ± SD)	37.53 ± 14.76	45.39 ± 13.15	0.01
BMI (Mean ± SD)	27.14 ± 4.71	29.62 ± 6.19	0.04
Race, n (%)			0.34
American Indian	0 (0%)	1 (2.3%)	
Black	10 (23.3%)	12 (27.3%)	
Asian	5 (11.6%)	1 (2.3%)	
White	18 (41.9%)	23 (52.3%)	
More than one race	1 (2.3%)	0 (0%)	
Latino	9 (20.9%)	7 (15.9%)	
Ethnicity, n (%)			0.57
Hispanic or Latino	11 (25.6%)	9 (20.5%)	
Not Hispanic or Latino	32 (74.4%)	35 (79.5%)	
Sex, Female, n (%)	34 (79.1%)	27 (61.4%)	0.71
Years of Education (Mean ± SD)	16.72 ± 2.40	15.70 ± 2.58	0.06
Asthma, n (%)	2 (4.7%)	23 (52.3%)	<0.001
Atopy, n (%)	13 (30.2%)	35 (79.5%)	<0.001
Polyp, n (%)		21 (47.7%)	

Abbreviations: Body Mass Index, BMI; ^a^ Unadjusted *p*-values.

**Table 2:** Comparison of sleep metrics calculated from actigraphy data of 44 CRS and 43 control participants, reporting Mean ± SD.

Sleep Metrics	Control	CRS	*p*-Value ^a^
Inter-daily Stability (IS)	0.46 ± 0.17	0.53 ± 0.16	0.06
Intra-daily Variability (IV)	0.74 ± 0.2	0.73 ± 0.23	0.8
Relative Amplitude (RA)	0.84 ± 0.19	0.88 ± 0.08	0.3
Lowest activity (L5)	21 ± 18.9	22.2 ± 20	0.8
Peak activity (M10)	308 ± 129	327 ± 113	0.5
Time in bed (TIB) (in minutes)	522 ± 109	495 ± 68.3	0.2
Total sleep time (TST) (in minutes)	446 ± 113	417 ± 65.2	0.2
Wake after sleep onset (WASO) (in minutes)	50.3 ±18.3	54.3 ± 24.1	0.4
Sleep efficiency (SE) (by %)	84.5 ± 5.02	84.1 ± 5.13	0.7

Abbreviations: Inter-daily Stability, IS; Inter-daily Variability, IV; Relative Amplitude, RA; Lowest activity, L5; Peak activity, M10; Sleep efficiency (by percent), SE; Time in bed (in minutes), TIB; Total sleep time (in minutes), TST; Wake after sleep onset (in minutes); WASO. ^a^ Two-sided *p*-value, level of significance at *p* < 0.05.

**Table 3:** Cognition score comparison between CRS and Control participants across six domains.

Cognitive Assessments	Control	CRS	Unadjusted *p*-Value ^a^	Adjusted *p*-Value
Episodic Memory	29.8 ±5.57	28.4 ±4.55	0.2	0.97
Processing Speed	92 ±14.7	82.9 ±12.5	0.003	0.23
Visuospatial Abilities	24.2 ±3.57	23.3 ±4.66	0.4	0.73
Semantic Memory	58.2 ± 11.2	53.8 ± 8.38	0.05	0.61
Working Memory	26.1 ± 5.16	24.8 ± 5.26	0.3	0.96
Executive Function	97.5 ± 17.5	88.1 ± 16.5	0.01	0.31

Abbreviations: Chronic Rhinosinusitis, CRS. ^a^ Regression analysis was used to compare cognition scores CRS participants to control participants, adjusting for age, sex, race, BMI, and years of education. Level of significance at *p* < 0.05. ^a^ Unadjusted *p*-values are also reported, level of significance at *p* < 0.05.

**Table 4:** Bivariate association testing in participants with CRS to determine the relationship between actigraphy-derived sleep metrics and cognition scores across six domains ^a^.

Predictor Variables	Episodic Memory	Processing Speed	Visuospatial Abilities	Semantic Memory	Working Memory	Executive Function
IS	0.12 (*p* = 0.5)	−0.03 (*p* = 0.8)	0.04 (*p* = 0.8)	0.08 (*p* = 0.6)	−0.09 (*p* = 0.5)	0.11 (*p* = 0.5)
IV	0.13 (*p* = 0.4)	0.02 (*p* = 0.9)	0.19 (*p* = 0.2)	0.18 (*p* = 0.2)	0.133 (*p* = 0.4)	−0.03 (*p* = 0.8)
RA	0.08 (*p* = 0.6)	0.18 (*p* = 0.2)	0.18 (*p* = 0.2)	0.04 (*p* = 0.8)	0.02 (*p* = 0.5)	−0.04 (*p* = 0.8)
L5	−0.11 (*p* = 0.5)	−0.13 (*p* = 0.4)	−0.17 (*p* = 0.3)	0.02 (*p* = 0.9)	−0.11 (*p* = 0.5)	0.17 (*p* = 0.3)
M10	−0.16 (*p* = 0.3)	0.02 (*p* = 0.9)	−0.03 (*p* = 0.8)	0.04 (*p* = 0.8)	0.03 (*p* = 0.9)	0.3 (*p* = 0.05)
WASO	−0.36 (*p* = 0.02)	−0.285 (*p* = 0.06)	−0.01 (*p* = 0.9)	−0.19 (*p* = 0.2)	0.2 (*p* = 0.2)	−0.01 (*p* = 0.9)
SE	0.26 (*p* = 0.08)	0.22 (*p* = 0.2)	−0.14 (*p* = 0.4)	0.1 (*p* = 0.5)	−0.16 (*p* = 0.3)	−0.04 (*p* = 0.8)

Abbreviations: Inter-daily Stability, IS; Inter-daily Variability, IV; Relative Amplitude, RA; Lowest activity, L5; Peak activity, M10; Sleep efficiency (by percent), SE; Time in bed (in minutes), TIB; Total sleep time (in minutes), TST; Wake after sleep onset (in minutes); WASO. ^a^ *p*-values adjusted for age, BMI, and years of education. Level of significance at *p* < 0.05.

## Data Availability

The data presented in this study is available upon request from the corresponding author.
